# Ditching Diet Talk: A Qualitative Study of Teachers Implementing Weight‐Inclusive Nutrition Curriculum in the High School Health Classroom

**DOI:** 10.1111/josh.70150

**Published:** 2026-04-13

**Authors:** Janet Gamble, Jordan Levinson, Deborah Hinchey, Katelyn Russell, Bernice Garnett, Lizzy Pope

**Affiliations:** ^1^ Department of Nutrition and Food Science University of Vermont Burlington Vermont USA; ^2^ Department of Biomedical and Health Sciences University of Vermont Burlington Vermont USA; ^3^ The Bromfield School Harvard Massachusetts USA; ^4^ College of Education and Social Services University of Vermont Burlington Vermont USA

**Keywords:** case study, high school curriculum, nutrition education, weight‐centric, weight‐inclusive

## Abstract

**Background:**

Nutrition education in U.S. high schools is traditionally taught from a weight‐centric approach. Emerging evidence suggests the need for alternative curricula that emphasize health behaviors rather than body weight. To address this gap, a weight‐inclusive nutrition (WIN) curriculum was collaboratively developed and implemented with high school health teachers.

**Methods:**

This pilot study examined the perspectives of three teachers using a small‐scale qualitative case study approach coupled with researcher‐led participatory action research. Interviews, teacher reflections, and classroom observation were used to triangulate data.

**Results:**

Findings revealed three overarching themes: challenges are inevitable, opportunities exist, and effective programing requires applicable design, support, and space to unpack embedded views. Eight sub‐themes further illustrated teachers' experiences and perceptions.

**Implications for School Health Policy, Practice, and Equity:**

Results highlight the need for sustained support systems and policy reform at local, state, and federal levels, as inequities related to food, bodies, and health extend beyond the classroom.

**Conclusions:**

Re‐framing nutrition education using a weight‐inclusive lens is possible; however, long‐term adoption depends on WIN specific support and collaboration within schools.

## 1 Introduction

Nutrition education is a key component of comprehensive health education in schools; it has the potential to positively impact student attitudes and outcomes towards food, bodies, and health well beyond the school years [1, 2, 3, 4]. Despite evidence that supports the long‐term positive impacts of nutrition education, the Centers for Disease Control and Prevention (CDC) reports that many U.S. students receive less than 8 h of required nutrition education each year [5], and Kenney et al. [6], note that in a sample of 117 kindergarten to 12th‐grade schools across the United States, 21.5% report not teaching about nutrition at all during the school year. Insufficient time and a lack of motivation, resources, expertise, and support have all been identified by schools and teaching staff as barriers to effective nutrition education programming and delivery [7, 8].

Furthermore, nutrition education has historically been taught from a weight‐centric viewpoint—one that centers on body weight as the primary determinant of health outcomes, attaches morality to food with “good” and “bad” labels, and suggests that weight‐loss and attainment of an ideal body shape improves overall health outcomes [9]. Weight‐centric approaches may mischaracterize the connection between body weight and health and have been associated with weight stigma, weight‐based bullying, and an increased risk of disordered eating behaviors [10, 11, 12]. Commonly implemented school‐based weight‐centric activities—including student weighing, food tracking, fitness testing and showing emotive films about food—may also be associated with increased shame, body dissatisfaction, anxiety, and disordered eating among students, with little evidence of corresponding health benefits [13]. Evidence also suggests that, among K‐12 students, weight‐based bullying and victimization are the most common forms of school‐based bullying [14], while Lin et al. [15], and Chen and Couturier [16] have reported that students in treatment for anorexia nervosa have identified school‐based nutrition education as being the trigger for the development of their eating disorder. Given the negative impacts that a weight‐centric approach may have on student health outcomes, a shift in the narrative around how food, bodies, and health are talked about in classrooms is needed. The weight‐inclusive framework challenges the dominant weight‐centric paradigm by arguing that health behaviors, not body size, are more impactful to health outcomes, that weight and health are profoundly influenced by the social determinants of health (SDOH), and that it is possible for people of all sizes to experience individual health [9]. Established weight‐inclusive concepts such as Health at Every Size and intuitive eating are embodied in this approach. These frameworks emphasize respect for body diversity, attunement to internal cues like hunger and fullness, and overall well‐being rather than weight loss. Several studies on weight‐inclusive interventions in adults have demonstrated that taking this approach may positively impact both physical and mental health outcomes while reducing the likelihood of anti‐fat bias and the development of disordered eating behaviors [17, 18, 19, 20, 21]. Weight‐inclusive *nutrition* (WIN) emerges when weight‐inclusive principles are paired with nutrition science.

Previous studies by Hinchey et al. [22, 23], which interviewed health educators and school health stakeholders, determined weight‐centric nutrition education values and activities dominate schools and health education classrooms. However, these studies also reported that schools and teaching staff expressed a desire for alternative nutrition curricula with the potential to reduce harm in their student populations. Based on these findings, and a dearth of knowledge and resources specific to WIN in schools, it was determined that there was a need to develop new WIN curriculum for schools and health teachers. Over the 2023–2024 school year and guided by researcher‐led participatory action research (PAR), an eight‐lesson WIN curriculum was developed and implemented in high schools in the northeast United States. Importantly, the curriculum was not based on any existing program but was intentionally designed by integrating WIN principles [24] (Table 1) with the National Health Education Standards (NHES) [25]. The purpose of this study was to better understand the challenges, opportunities, and key components identified by classroom health teachers in the development and implementation of a novel WIN curriculum.

**TABLE 1:** Guiding principles of weight‐inclusive nutrition [24].

**Centers a foundation of nutrition science**
Focuses on building understanding of macro and micronutrient function, sources, and recommended intake
**Analyzes the relationship between nutrition and health outcomes**
Counters misinformation about weight and health
Integrates the social determinants of health
Examines the harm stemming from disordered eating and eating disorders
**Examines nutrition information critically**
Teaches skills to find and interpret nutrition information that is reliable, evidence‐based, and created by experts
Respects an individual's own context and knowledge of their body
**Promotes attuned and joyful eating practices**
Emphasizes attunement to hunger and fullness cues
Recognizes the benefits of eating for pleasure and nourishing one's body with a variety of foods
**Emphasizes the impact of sociocultural influences on relationships with food**
Acknowledges the cultural, systemic, socioeconomic, familial, and peer influences on food choice
Challenges the assumptions of diet culture
Addresses the existence and health impacts of anti‐fat bias

## 2 Methods

This pilot study used an exploratory, single case‐study design coupled with researcher‐led PAR. Combining case‐study design with PAR allows for a more detailed examination of a specific phenomenon or situation while also ensuring that the research is relevant and meaningful to the participants. For this study, a common case was defined and bounded [26] as the development and piloting of WIN curriculum over the 2023–2024 school year. Working collaboratively with three high‐school health education teachers (hereafter referred to as Teacher A, B, or C) allowed for greater knowledge production in addressing the real‐life issues faced by teachers and students [27] when focusing on contextual issues around food, bodies, and health.

### 2.1 Participants

Teachers A, B, and C were identified as potential participants for the current study through purposeful sampling using existing professional networks and were contacted by the lead researcher to assess interest. Inclusion criteria included being a high school health educator in the northeastern United States. Teachers did not need to have familiarity or experience teaching from a WIN perspective to participate.

### 2.2 Instrumentation

Initial lesson development occurred in Fall of 2023. Over an eight‐week period, the lead researcher worked closely with Teacher C to develop the curriculum, which was designed in alignment with the 2024 NHES [25]. Each of the eight WIN lessons corresponds to a specific standard, with performance indicators mapped to guiding WIN principles. Developed by the research team, WIN principles aim to pair basic nutrition concepts and skills to individual eating goals while acknowledging the dynamic interaction that exists between food, bodies, and health (Table 1) [24]. Consistent with a PAR approach, curriculum development was iterative and collaborative: the researcher outlined WIN‐focused lessons and learning objectives, while Teacher C contributed classroom‐appropriate assessments, activities, and instructional materials to achieve shared learning outcomes using a backward design approach [28]. Following completion of the first curriculum iteration, the lessons were shared with Teachers A and B for implementation across all sites during Spring of 2024. Teacher A first piloted the lessons, then teacher B, and finally teacher C. Lessons were modified as feedback was received from teachers, meaning that several of the lessons took a slightly different form for Teacher A than Teacher C. Lesson overviews are provided in Table 2.

**TABLE 2:** Summary of WIN education lessons.

National health educationstandard	Weight‐inclusivenutrition lesson	Performance indicators & learning targets	Lesson activities
Use functional health information to support health and well‐being.	*What Is Nutrition?* *‐Macronutrients* In this lesson students learn about the essential nutrients needed to promote health and well‐being. Students identify key macronutrients the body needs as well as examples of what foods these nutrients are found in.	1.12.3 Evaluate behaviors that reduce or prevent illnesses and injuries. 1.12.4 Evaluate practices and behaviors that support health and well‐being, including how to manage health conditions. ∼ 1. I can use strategies such as identifying the macronutrients found in food to evaluate how my body uses those, what foods I find those nutrients in, and why they are important to my overall nutrition and health.	1: Prior nutrition knowledge elicitation: warm‐up discussion. 2: Guided note‐taking worksheet. 3: Small‐group macronutrient classification challenge.
Advocate to promote health and well‐being of self and others.	*What is Diet Culture?* In this lesson students learn about diet culture and create strategies to support positive health and well‐being. Students identify where they see diet culture and recommend strategies that advocate for their own health and well‐being as well as that of others in their community.	8.12.4 Demonstrate self‐advocacy and skills and strategies to promote health and well‐being. 8.12.5 Demonstrate advocacy skills and strategies to promote health and well‐being at interpersonal, community, societal and environmental levels. ∼ 1. I can describe how I may be influenced by such things as diet culture and anti‐fat bias and demonstrate self‐advocacy skills that center my own knowledge to improve my health and well‐being. 2. I can demonstrate advocacy skills and strategies that acknowledge the cultural norms that influence health and well‐being, such as diet culture and anti‐fat bias, at interpersonal, community, and societal levels.	1: Exploration of students' initial understandings of diet culture using sensory descriptions (what it looks like, sounds like, and feels like). 2: Small‐group classification and discussion of diet culture examples (diet culture or not?). 3: Collaborative identification of diet culture manifestations and completion of a school culture worksheet.
Analyze influences that affect health and well‐being.	*What Influences My Food Choices?* In this lesson students learn about factors that affect their food choices. Students analyze how internal and external factors can influence food choice. Students work together to come up with solutions for improving health equity in their community.	2.12.2 Evaluate how social determinants of health influence health behaviors, health outcomes and health equity. 2.12.3 Evaluate how individual, interpersonal, community, societal, and environmental influences and factors affect health equity. ∼ 1. I can provide a rationale how factors such as income, education, the physical environment, culture and social networks influence food choices and nutrition status. 2. I can evaluate health inequity in my community and develop a solution to promote health and well‐being	1: Classification of food choices as internally or externally influenced. 2: Discussion of factors affecting equitable access to food and health. 3: Development of community‐based solutions addressing food and health inequities.
Access valid and reliable resources to support health and well‐being.	*Where Can I Find Reliable Food and Nutrition Information?* In this lesson students learn how to access reliable food and nutrition information that promotes health and well‐being. Students identify food‐related resources (i.e., social media, books, podcasts) that promote positive food experiences and how to apply strategies to manage misinformation.	3.12.4 Use valid and reliable sources of health information, products, services, and other resources. 3.12.5 Apply strategies to manage misinformation and disinformation. ∼ 1. I can find, gather, and interpret valid and reliable sources of food and nutrition information, products, and services that do not prioritize weight as the main determinant of my health 2. I can apply strategies to investigate expert vs. non expert voices when managing food and nutrition information while recognizing that my needs will be different than others	1: Evaluation of social media content (reliable or not?) using a checklist. 2: Creation of a digital poster promoting reliable food and nutrition information.
Use interpersonal communication skills to support health and well‐being.	*Talking to Others About Food and Nutrition*. In this lesson students learn how to effectively communicate messages about food and nutrition that promote health and well‐being. Students identify key interpersonal communication strategies for talking about food and nutrition that supports the food choices of themselves and others.	4.12.1 Apply effective communication skills across multiple modes of communication and media formats to support health and well‐being of self and others. 4.12.3 Demonstrate how to ask for and offer assistance to support the health of self and others. ∼ 1. I can use effective communication skills that are not judgmental towards people with different body types across multiple modes of communication and media formats to support the food choices of myself and others. 2. I can demonstrate how to ask for food and nutrition information and offer assistance to support the health of myself and others while recognizing that nutrition information is often based on diet culture.	1: Identification of interpersonal communication skills. 2: Small‐group exploration of alternative language use to promote positive language around food, bodies, and health. 3: Reflection on negative language around food, bodies, and health, and formulation of positive responses outside of class.
Demonstrate practices and behaviors to support health and well‐being.	*Nourishing My Body in a Gentle Manner*. In this lesson students learn about building a healthy relationship with food. Students identify that factors such as hunger and fullness cues influence eating behaviors. Students learn about trusting their bodies to make food choices that feel good for them.	7.12.3 Adapt practices and behaviors to support individual and collective health and well‐being. 7.12.4 Demonstrates a variety of practices and behaviors supporting individual and collective health and well‐being. ∼ 1. I can demonstrate a variety of practices and behaviors, such as identifying hunger and fullness cues and eating for pleasure, that support the health and well‐being of myself and others. ∼ 2. As my life circumstances change, I can recognize that my overall food and nutrition goals may change, and I am able to modify strategies to meet these while continuing to nourish my body in gentle manner.	1: Viewing of introductory video on physiological hunger and fullness responses with accompanying worksheet. 2: Introduction to and self‐assessment using the Hunger/Fullness scale. 3: Mindful eating practice engaging all five senses.
Use a decision‐making process to support personal and community health and well‐being.	*Making Decisions About Food and Nutrition*. In this lesson students learn effective ways to create a personalized nutrition plan that meets their individual needs. Students design an individual eating plan that is based on such factors as personal preference, food access and availability and physical and emotional needs.	5.12.4 Analyze a variety of options based on priorities and potential outcomes when making a health‐related decision. 5.12.6 Develop a plan of action to implement a health‐related decision. **∼** 1. I can prioritize alternatives when designing and implementing an eating plan that provides my body with the energy it needs to move in a way that feels good and meets my individual needs	1: Creation of a word cloud illustrating students' reasons for eating. 2: Case study analysis of daily food choices and associated barriers. 3: Development of a personalized daily nutrition plan using a “A Day in the Life of ___?” framework.
Use a goal‐setting process to support health and well‐being.	*Setting My Own Personalized Food and Nutrition Goals*. In this lesson students learn how to use goal setting skills around food and nutrition. Students identify how to set food and nutrition goals that support their own individual health and well‐being. Students discuss facilitators and barriers that might influence their ability to attain their goals and come up with strategies to address these.	6.12.4 Implement a plan that addresses supports and barriers to attaining a health‐related goal. 6.12.6 Evaluate the goal‐setting process and outcomes on health and well‐being. ∼ 1. I can implement a plan that acknowledges that there are facilitators and barriers to how I eat. 2. I can evaluate a goal‐setting process and outcomes using food and nutrition that nourishes my body in a gentle manner and meets my personal health and well‐being goals. 3. I can do this while recognizing that hunger and fullness cues and eating for pleasure (without shame or guilt) may influence my ability to attain my own personal food and nutrition goals.	1: Paired identification and understanding of SMART goals. 2: Small‐group practice in setting gentle food and nutrition SMART goals. 3: Individual development of personalized gentle food and nutrition SMART goals

In addition to the curriculum, multiple data collection instruments were developed to capture teachers' perceptions and experiences throughout the study. Drawing on relevant literature, previous research and the interdisciplinary expertise of the research team (nutrition, eating disorders, psychology, public health, community schools), semi‐structured pre‐ and post‐interview questions were developed in addition to a six‐item post‐lesson teacher reflection (see Supporting Information). An observation guide was designed to document instructional practices and classroom dynamics during lesson implementation (see Supporting Information). Finally, the lead researcher kept a journal throughout the study to engage in reflexive practice related to the research process.

### 2.3 Procedure

Following Institutional Review Board approval [CHRBSS (Behavioral): STUDY00002892, approved January 16, 2024], Teachers A, B, and C were contacted via email by the lead researcher. All participants were provided with a research information sheet which outlined risks, benefits, confidentiality, voluntary participation, and compensation. Each teacher was compensated $250 for their participation. Letters of institutional support were acquired from each participating school, acknowledging that members of the research team would be present in classrooms for observation.

Each participant was responsible for implementing the lessons to the best of their ability without receiving prior training in the curriculum. Interviews were conducted with each teacher before curriculum implementation and again after all lessons had been delivered. These sessions lasted approximately 30–45 min and took place either in person or via Microsoft Teams [29]. All interviews were recorded and transcribed using the Google Recorder app [30]. Transcripts were cleaned by two undergraduate students and reviewed by the lead researcher. In addition, teachers completed written reflections following each lesson using the pre‐defined set of questions. Lesson observations were conducted by two members of the research team either in person or via Microsoft Teams [29]. Lessons were not recorded; rather, the lead researcher took detailed notes about each lesson using the observation guide.

It is important to note that while a shared curricular framework guided instruction, teachers implemented evolving versions of the lessons as iterative revisions were made based on participant feedback. As such, fidelity of implementation varied across sites due to differences in timing, classroom dynamics, and instructional constraints. These contextual factors, along with the researchers' positionality as white female scholars committed to a weight‐inclusive perspective, were carefully considered during analysis by focusing on implementation processes rather than cross‐site comparison and by engaging in reflexive discussion to examine potential interpretive bias.

### 2.4 Data Analysis

Following Creswell and Poth's data analysis spiral [31], analysis proceeded through cyclical phases of reading, memoing, coding, classifying, interpreting, and representing the data. During first‐cycle coding, the research team engaged in an iterative process of open coding to identify developing concepts [32]. Three team members independently coded a sample transcript (*n* = 10 pages), generating 18 initial codes. The team met to discuss discrepancies, refine code definitions, and finalize a codebook of 17 codes (see Supporting Information), which was then applied to all transcripts. Through pattern coding and constant comparison, two team members independently analyzed the coded data, grouping excerpts into broader categories aligned with the spiral's interpretive phase [31]. Weekly meetings were used to resolve coding discrepancies through negotiated consensus, before refining categories into three overarching themes and eight sub‐themes. Observational field notes were analyzed separately using analytic memoing and integrated during interpretation to triangulate teacher‐reported experiences with observed classroom dynamics [33]. Member checking [34] and peer debriefing [35] were used throughout the process to ensure transparency. Dedoose qualitative data analysis software was used to analyze all data [36].

## 3 Results

**FIGURE 1:**
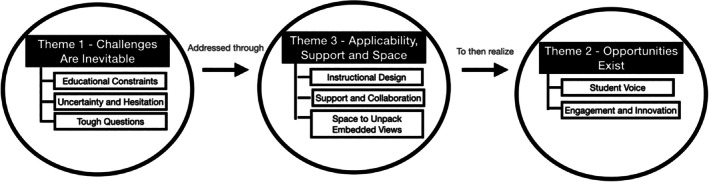
Relationship of themes and sub‐themes.

Table 3 summarizes the professional backgrounds of the participating health teachers and the demographic characteristics of each school. The three teachers, who were all white females, varied in prior familiarity and experience with WIN principles, years of teaching, and grade levels taught, while schools differed in size and student demographics. Data analysis yielded three themes and eight sub‐themes that emerged retrospectively. Figure 1 details how the themes and subthemes relate to each other conceptually. Theme 1 (challenges) captures the inevitable obstacles teachers encountered during WIN curriculum implementation. Theme 3 (applicability, support, and space) represents practical strategies and conditions that helped teachers navigate these challenges. When such supports were present, Theme 2 (opportunities) emerged, reflecting the ways teachers could transform challenges to enact meaningful instructional practices. In this way, Theme 3 serves as a bridge between the macro‐level challenges (Theme 1) and opportunities (Theme 2), showing how practical supports helped teachers navigate the challenges and leverage opportunities during curriculum implementation. Themes, sub‐themes, and illustrative quotes are presented in Table 4. With the exception of sub‐theme 1.3 (tough questions), all teachers reported all themes regardless of their individual school context as described in Table 3. This exception may reflect that students at school B were relatively advanced, potentially due to attending a more resourced school with a teacher who had prior experience implementing WIN principles.

**TABLE 3:** Participant Experience and School Demographics^a^.

School/teacher	Familiar with WIN principles	Prior teaching using WIN principles	# of years teaching health education	Classroom enrollment (# students)	Grade(s) taught	School size (# students)	% of students qualified for free and reduced lunch^b^	% of students of the global majority^c^
A	No	No	20	15	9 & 10	897	56	42
B	Yes	Yes	9	20	10	327	11	30
C	Yes	No	25	20	11 & 12	725	35	19

^a^ Data is from 23/24 academic school year.

^b^ Percent free and reduced lunch is used to measure the concentration of low‐income students at a school [37].

^c^ Percent global majority measures the number of students who do not identify as Caucasian [38].

**TABLE 4:** Themes and illustrative quotes.

Theme	Sub theme and supporting quotes
**1. Challenges**	**1.1 Educational Constraints**
	**Quote 1**: It's hard to get into the nitty gritty of it [nutrition education] which would be great so they could better understand what this food is actually doing to them, because we just don't have the time. (*Teacher A*) **Quote 2**: Because it's such a big conversation [weight‐inclusive nutrition] and again with the few lessons that I have with them, it's not a ton of time to get into it. (*Teacher B*) **Quote 3**: My big issue is always time since our periods are so short. (*Teacher C*) **Quote 4**: So collaborating… there's just never been anybody to collaborate with. (*Teacher C*) **Quote 5**: I feel like I have been trying to cobble something together [weight‐inclusive materials]… nutrition specific I've kind of cobbled together outside of the conferences. (*Teacher B*) **Quote 6**: And ideally they [the lessons] could really fit in my class time if I didn't have a highly distracted class. (*Teacher A*) **Quote 7**: It's that fine balance, to hold kids accountable at my school for doing something, but also giving them the space to do activities when they don't want to. (*Teacher C*)
	**1.2 Uncertainty and Hesitation**
	**Quote 1**: I just want to make sure that I do it [weight‐inclusive nutrition education] well… it's just making sure that I do it with validity. It's something different than what I am used to doing. (*Teacher A*) **Quote 2**: And without having enough experience, how do I make the connections? (*Teacher C*) **Quote 3**: I'm talking about [WIN] with my kids and my husband, but I wasn't doing it in school because I'm afraid—I used to say this to the kids, I am terrified of giving you all a complex about food and making this a really negative experience. I don't want to do that. But I just kind of left it as like I don't want to do it, and then would just talk about the science and not really make it practical. (*Teacher B*)
	**1.3 Tough Questions**
	**Quote 1**: I had a long conversation with one class about body size and health. They asked questions about moderation and self‐control and were very concerned that if they didn't label foods as “good” or “bad” then they would only eat things like cake. I tried to prompt them and ask them, “Do you think your body only wants cake?” (*Teacher B*) **Quote 2**: My other classes said, “Well, but some foods *are* good and some foods *are* bad.” (*Teacher B*) **Quote 3**: They mentioned that heart disease was caused by body size (*Teacher B*)
**2. Opportunities**	**2.1 Student Voice**
	**Quote 1**: And some students have pushed back in PE because of writing out their own meal plan for a day. It's a trigger. (*Teacher C*) **Quote 2**: It's a small percentage but every semester there's one or two that will come to me and say I can't do this assignment. It's triggering for me. Is there something different I can do? (*Teacher A*) **Quote 3**: It was nice to read some of their course evaluation feedback because many of them talked about weight inclusivity and how much they get out of it, which was awesome. (*Teacher B*) **Quote 4**: I told you I had that last student that was like, you should definitely teach this one next year, instead of the one that you taught earlier. It was awesome. They'd never talk about this stuff. You address a topic head on that they don't talk about and they loved talking about it. (*Teacher B*)
	**2.2 Engagement and Innovation**
	**Quote 1**: I mean a place to add on to it, if you really want to like hit all the health skills which we could talk about, like advocacy, which is already kind of in there with the diet culture one, so that could be more of like a direct connection between the curriculum and the national skills. So then some of the focus gets taken away from the content and onto the skill, which is fine because you need the skill to do well in nutrition. (*Teacher B*) **Quote 2**: Skills was a huge thing in health. So like thinking about nutrition and how that might play a role in the content piece of it because we're so content driven sometimes that we're not also always focusing on skill related stuff that would be more applicable. I know other teachers across [the] County are trying to get to more skills based and I know that that is something that I would like to try to pursue more. (*Teacher A*) **Quote 3**: You know my underlying thinking, has honestly, just been like, strengthened and reinforced. This has just given me more resolve, more evidence, more support to disconnect weight and health even further. It's already to me, it was already disconnected. But having the experience of talking to high school students so directly about weight inclusiveness, right? Like not as an aside, or as a part of, but just like really addressing it head on has just made everything stronger for me. (*Teacher B*)
**3. Effective WIN Programming**	**3.1 Instructional Design**
	**Quote 1**: I thought the “reasons why we eat” lesson went well. The students were able to think past hunger signals and likes/dislikes. This set them up to think more deeply about external factors that could impact access to food and health. (*Teacher B*) **Quote 2**: The activity that the students did to identify images as diet culture or not in my opinion went very well. It took some groups a minute to get going, but when they did they were having great conversations that led to a great class discussion with me pushing them to think about this in different ways. (*Teacher A*) **Quote 3**: So my question would be, what do I need to do to incorporate not just the idea of weight inclusivity but also around how to plan meals, like the planning piece? How can I incorporate it in to talk about the ideas but then give them something tangible besides the mindset of it? So that they can take it with them and make choices throughout the day. (*Teacher C*) **Quote 4**: Maybe having a couple different case studies that students could work through in small groups. Having different perspectives. Groups could report out on their case study with questions that were unique to their person. (*Teacher A*)
	**3.2 WIN Specific Support and Collaboration**
	**Quote 1**: I think this requires some training on how to respond when someone says something offensive. Our school has done a lot of training around how to respond to microaggressions and how to be an “upstander,” and I'm able to apply that training a lot in the classroom. If a teacher is newer or their school hasn't held that type of training, they made need more support in how to facilitate difficult conversations. (*Teacher B*) **Quote 2**: I like the idea for newbies, taking each lesson, and creating… like applying three or four of the lessons into an activity, whether it's goal setting, whether it's a self‐reflective piece. And I know that's just big picture thinking but for them [teachers] to be able to take the ideas and to apply them for themselves first. (*Teacher C*) **Quote 3**: I love the idea of having coursework, even if it's an 8‐h coursework of a couple of articles where you have to read to build that foundation, so that I understand the language like the hunger scale. (*Teacher C*) **Quote 4**: It might be a good idea to add in some FAQs or examples of language that teachers can use to question students in that way. (*Teacher B*) **Quote 5**: And I think those types of workshops where we get to kind of work with our colleagues across the state helps, because I might be like, oh cool. Like I want to try that. You know like just having different ideas. Yeah, I think that's valuable when we can get together and collaborate a little bit on things. (*Teacher A*) **Quote 6**: If I'm going to use these again, which I would like to and go through these things again, maybe I might need more information. I think having a workshop that focuses on diet culture and being able to go through this lesson with colleagues (meaning health educators) could be helpful when we actually need to teach this in the class. (*Teacher A*) **Quote 7**: Add one or two resources about the hunger scale. I would like more information on diet culture. Resources on equity. (*Teacher C*) **Quote 8**: How to respond when kids say that foods are good or bad. I also think that PD to help with the hard questions about body weight, nutrition, and health, would be helpful. (*Teacher B*)
	**3.3 Space to Unpack Embedded Views**
	**Quote 1**: In my head, I understand. And I've always struggled with eating. So a majority of the time before the last 2 years, my idea of eating was not eating so that I could try to lose weight. And I am just learning that doesn't work. So this is really challenging my own self. So the acceptance, I'm not there yet. But I see the benefits—kind of. If that makes sense. For me—that's nothing to do with kids. (*Teacher C*) **Quote 2**: So, having that structure that you built into the curriculum was super helpful… because this is something I think about in my own life. Like, how do I promote nutrient density? How do I try to nourish my body the best I can, and also not be super restrictive? And also for pleasure, right? Like, I'm always trying to hold those things and think about them. (*Teacher B*) **Quote 3**: However, they are still susceptible to misinformation even with this skill [finding reliable information]. I asked them in class what they believe that they see on social media and they came up with so many things—that apple cider vinegar helps you lose weight, that Khloe Kardashian's supplements look like they work, etc. In the classroom, they are able to think critically but there is something that is not carrying over to their phones and life outside of the classroom. (*Teacher B*) **Quote 4**: There's no perfect. There's no ideal shape. There's no ideal this [diet]. It's like decoupling that a little bit, right? It [the curriculum] was a way for me to articulate to the kids that nutrition is still really important. It's like the conversation I'm thinking of with a parent. Like just because we're not putting value statements on food, doesn't mean that it's not important. It's still important to nourish yourself but we're doing it in a way that feels sane and comfortable and nice and not harmful. (*Teacher B*)

### 3.1 Theme 1: Challenges Are Inevitable as the Narrative Around Food, Bodies, and Health Shifts

This theme describes challenges that teachers faced during the creation and implementation of the WIN curriculum. While many of these were related to limited classroom resources, discussing nutrition independent of body weight created a unique set of challenges.

#### 3.1.1 Sub‐theme 1.1—Educational Constraints

Generally, teachers acknowledged that they were affected by time constraints (Quotes 1–3), a lack of access to professional development (“Personally, I would be happy with any professional development in the realm of nutrition” Teacher A), and minimal opportunities to work with others (Quote 4). Procuring WIN education resources specifically was also identified as a challenge (Quote 5). Findings from researcher observations support that classroom dynamics (Quote 6 and 7), including grade level, time of year (“This is the time of year when I need to teach standing on my head to keep everyone engaged” Teacher B), number of English language learner (EL) students, and physical space can also affect the ability of teachers to effectively implement nutrition lessons.

#### 3.1.2 Sub‐theme 1.2—Uncertainty and Hesitation

Shifting the way in which one teaches about food, bodies, and health posed unique challenges for all teachers. Those with limited experience teaching from this paradigm expressed concern about their lack of understanding (Quotes 1 and 2), and while it was acknowledged that this hesitation is natural, it could also serve as a barrier to implementation for teachers less familiar with the paradigm (“I felt an underlying current of fear when I asked my students to imagine permitting themselves to eat whatever they wanted. I think other teachers might feel the same and that could be a barrier to implementation” (Teacher B)). For others, it was less about being comfortable with the material and more about not knowing how to approach it (“For me personally, I think it's more that I don't know how to approach it. I don't think it's necessarily me being uncomfortable, because there's a lot of things out there that I talk about that could be uncomfortable” Teacher A). It was further suggested that teaching about food, bodies, and health from this perspective can be challenging even for those with experience, sometimes leading teachers to avoid the topic altogether (Quote 3).

#### 3.1.3 Sub‐theme 1.3—Tough Questions

Teaching about food, bodies, and health independent of weight posed additional challenges for Teacher B specifically. They reported that students were curious about the nuances of weight‐inclusive principles and often asked questions that challenged the paradigm. The idea that there aren't any “good” foods or “bad” foods was difficult for some students to accept and they expressed that removing these labels may lead to a loss of control around food (Quote 1). Other students argued that there are indeed “good” foods and “bad” foods and that it is not possible to speak of foods without labels (Quote 2). Teacher B also indicated that students struggled with the idea that body weight is influenced by many factors other than calorie intake alone (“They were also very stuck in the “calories in = calories out” paradigm. They asked questions like “but won't you gain weight if you eat too much?”), and that they raised concerns that this paradigm fails to identify that certain chronic disease states are caused by body size (Quote 4).

### 3.2 Theme 2: Opportunities Exist to Engage in Nutrition Learning That is Skills‐Based, Respectful, and Supportive of Individual Student Needs

This theme highlights that teaching from a WIN perspective provides opportunities for both students and teachers to engage in learning that is respectful and supportive of individual needs. Student voice emerged as a powerful tool, enabling teachers to engage more deeply with learners and foster an environment that prioritizes the development of practical food and nutrition skills that are applicable to real‐world situations.

#### 3.2.1 Sub‐theme 2.1—Student Voice

Providing students with the opportunity to share perspectives, experiences, and opinions around food, bodies, and health played an important role in supporting students in the learning environment and educational process. Teachers at two of the schools identified that students had previously spoken to them about nutrition education being a trigger (Quotes 1 and 2), while it was also noted that some students were questioning current nutrition education practices (“And I have heard from students in the past, wanting you know, this more like weight‐inclusive kind of thing and you know, just because a person looks a certain way doesn't mean they're necessarily unhealthy… So I've heard the needs of that” Teacher A). Teachers also shared that some students vocalized that this new approach to talking about food, bodies and health resonated with them and the conversations were more meaningful (Quotes 3 and 4).

#### 3.2.2 Sub‐theme 2.2—Engagement and Innovation

WIN was seen as an opportunity by teachers to do something new and different that would add value to their students' learning (“I'm excited about teaching them a different way. Because I have investment in all of them… I want to make an impact on my students…I want to see their success and to see them learn something, to find value” Teacher C). It was also recognized as an opportunity to replace content‐based nutrition education with skills‐based nutrition education by applying key WIN principles—such as advocacy—to the lessons (Quotes 1 and 2). Inspiration was found from the lessons that reinforced teachers' beliefs that there is an alternative way to educate students about food, bodies, and health (Quote 3).

### 3.3 Theme 3: Effective WIN Programming Requires Applicable Instructional Design, Adequate Support and Collaboration, and Space to Unpack Embedded Views Across Health Teachers and Students

This theme encapsulates the multiple aspects that must be considered for effective WIN curriculum design and implementation. Instructional design, support and collaboration, as well as having space to unpack embedded views about food, bodies, and health, were identified as key components.

#### 3.3.1 Sub‐theme 3.1—Instructional Design

Creating engaging and impactful learning experiences was crucial to ensure that teachers and students got the most out of the curriculum. Teachers were able to assist the research team in understanding when a lesson was effective and why (Quotes 1 and 2) and provide suggestions on how to improve when needed (Quotes 3 and 4). Modeling activities for students (“I think the identifying what's reliable or not activity is a good one, but what made it hard for the students was they didn't have a model so going through it with the checklist was a bit challenging for them I feel at first” Teacher A) and having opportunities to practice application of knowledge (“But thinking, like how would it [the lesson] be most applicable to a student?” Teacher C) were considered essential for student success when working in this paradigm.

#### 3.3.2 Sub‐theme 3.2—WIN‐Specific Support and Collaboration

Having support specific to WIN principles was considered an important piece for effective implementation of the curriculum. Specifically, adequate resources for teachers new to this paradigm were mentioned (Quotes 1 and 2). Teachers also identified the need for resources that address how to effectively incorporate WIN language into conversations with students (Quotes 3 and 4). Workshops and colleague collaboration were also recognized as being an essential part of feeling confident with this curriculum (Quotes 5 and 6), and resources related to specific WIN topics such as diet culture, intuitive eating, Health At Every Size, and weight‐stigma were also requested (“HAES, intuitive eating, weight stigma—I think teachers need to have an understanding of *why* foods aren't good or bad and why body weight is a terrible indicator of health” Teacher B, Quotes 7 and 8).

#### 3.3.3 Sub‐theme 3.3—Space to Unpack Embedded Views

Personal experience with food, bodies, and health requires time and space to unlearn and unpack the complex and often deeply ingrained ways they are experienced in society. Each teacher admitted that they were on their own journey with this unpacking and unlearning of embedded views alongside their students as the new curriculum was utilized (“I can see why we need to do this, why students need it, but I have to unlearn” Teacher A), (Quotes 1 and 2). The teachers also commented on how students experienced this unlearning of standard practices along a continuum. Some students appeared to struggle with this shift in thinking (Quote 4) while others were able to alter the way in which they used language around food, bodies and health in a more positive manner (“But I just remember her [a student] saying ‘healthier’. Like this is a ‘healthier’ option. And I said could you actually use different language and take healthier out? How else could you say what you want to say without using that word? And then, I don't remember what her response was exactly, but I do remember her changing it. And me thinking oh there you go, good job. Yeah, I like that” Teacher A). The importance of holding time and space to reexamine and deconstruct commonly accepted practices in the classroom was also noted (Quote 5).

## 4 Discussion

This exploratory study examined the critical elements associated with the development and adoption of a WIN curriculum in high‐school health classrooms in the northeast United States. Through the process of researcher‐led PAR, three main themes were developed from the data: (1) challenges are inevitable as the narrative around food, bodies and health shifts; (2) opportunities exist to engage in nutrition learning that is skills‐based, respectful and supportive of individual student needs; and (3) effective WIN programming requires applicable instructional design, adequate support and collaboration, and space to unpack embedded views for both health teachers and students. Our findings suggest that WIN implementation can be understood as a process where inevitable challenges (Theme 1) must be addressed through specific design and support components (Theme 3) to realize the potential opportunities (Theme 2) for student‐centered learning (Figure 1). These preliminary findings offer insight into the realities of classroom implementation, as well as the supports needed to make such a paradigm shift sustainable and effective. The eight‐lesson WIN curriculum was shaped through a collaborative approach with three educators, reflecting both the core WIN principles and the realities of educating high school students. Involving teachers as co‐researchers allowed their needs and perspectives to directly inform curricular content and implementation strategies, consistent with PAR's emphasis on collaboration and shared decision‐making. For example, throughout the iterative action research cycles, we centered active learning techniques, and the co‐creation process shaped outcomes by creating a sense of respect and trust between teachers and the university research team.

Prominent challenges identified by all participating teachers, and supported in the literature, include lack of time, support (funding, resources, adequate nutrition training), classroom management (poverty, chronic absenteeism, anxiety and depression, cellphone policies), and navigating stakeholder involvement (school culture, administration, parents/guardians) [39, 40, 41]. This, compounded with a lack of resources tailored to WIN content can undermine the flexibility and ongoing support that teachers need to implement new curriculum. These practical barriers were intensified by uncertainty and hesitation in teaching from a weight‐inclusive perspective, especially among educators new to the approach. This hesitation, while natural, reflects a broader tension in education where shifting long‐held beliefs can provoke discomfort or confusion—even among teachers well‐versed with the content [42, 43]. Additionally, student engagement with the topic added complexity to the situation. While the questions from students demonstrated curiosity about the relevance of the material, they also revealed entrenched beliefs about “good” and “bad” foods, body size, and personal responsibility for health—ideas that are regularly reinforced by societal and cultural norms, as well as scientific research and the medical field [10, 44, 45]. Teachers were therefore required to navigate their own learning curve, influenced by personal experiences with food, bodies, and health, while also facilitating critical discussions with students encountering weight‐inclusive principles for the first time and asking important questions about health behaviors and/or weight and health. These initial findings highlight that while WIN curriculum is promising, its implementation demands adequate and intentional support structures, including built‐in curriculum resources like teacher notes and guides for answering difficult questions that may emerge from students, as well as more extensive professional development and administrative backing [46, 47].

Additionally, as a case study emphasizing contextualized understanding of the development and piloting of the WIN curriculum, this research examined how implementation unfolded within real school settings. Rather than treating the challenges identified as inherent to the curriculum itself, the case study approach highlighted how student beliefs, prior messaging about food, bodies, and health, and existing instructional routines shaped tensions during implementation. Attending to these contextual factors across schools clarifies why challenges emerged and how they unfolded in practice.

Results also revealed opportunities to elevate student voice and shift towards more meaningful, skills‐based learning. Teachers shared that creating space for students to share personal experiences and critiques of traditional nutrition education appeared to enhance engagement and give them confidence to speak openly about their own experiences with food, bodies and health. As Teacher A commented to the lead researcher following the lesson on diet culture “the students were engaged… they don't want to talk about MyPlate, this is what they've been asking for.” This aligns with the literature that emphasizes the importance of student‐centered approaches in health education, particularly when addressing sensitive topics [48, 49, 50]. The WIN approach may also serve as a catalyst for pedagogical innovation. Teachers described how they saw the curriculum as an opportunity to move beyond static, content‐heavy frameworks towards more dynamic, skills‐based education rooted in critical thinking and real‐world application. Acting on this feedback, the research team worked collaboratively with Teacher B to create a comprehensive guide on adapting the WIN lessons to each of the NHES standards using the skill of advocacy. This mirrors calls within health education scholarship to prioritize skills‐based competencies in the classroom [25, 51]. Importantly, teachers reported feeling energized by the opportunity to try something different without having to develop a curriculum from the ground up. Together, these preliminary findings suggest that WIN education not only addresses important gaps in content but may also create pedagogical conditions that support deeper student engagement and reflection around food, bodies, and health.

The successful implementation of a WIN curriculum hinges not only on teacher willingness but also on intentional design and the presence of suitable support and collaboration. Teachers stressed the importance of thoughtful instructional design, and it was observed that those lessons that included opportunities for students to model behaviors and practice skills—such as how to talk to others about food, bodies and health—were the most effective and engaging. This aligns with best practices in health education, which advocates for experiential, student‐centered learning to promote long‐term skill retention and behavior change [52, 53, 54]. Feedback from teachers was also instrumental in ensuring that lessons met students where they were developmentally. Beyond lesson design, teachers identified the need for greater access to WIN‐specific resources and professional development, similar to what is acknowledged in the literature [40, 41] to build their own confidence and competence. This request reinforces the idea that shifting paradigms in health education requires more than content—it requires a supportive network of partners who have the power to influence curricular content and adoption [23]. Finally, all teachers acknowledged the need for space to unpack embedded views—for themselves and their students—as long‐held assumptions about food, bodies and health were challenged. These experiences, while sometimes uncomfortable, underscore the importance of creating space in classrooms and in professional development for critical reflection and unlearning of deeply personal health beliefs. Collectively, these components illuminate that effective WIN implementation is not a plug‐and‐play process; rather it is an iterative, relational, and context‐sensitive endeavor.

Implementing WIN education is undoubtedly complex. However, given the persistent limitations of traditional nutrition education in addressing disordered eating and weight‐based bullying [55], there is a compelling case for trying something different. It is important to acknowledge that discomfort and uncertainty are natural parts of this paradigm shift, and that teachers do not need to have all the answers to begin the work. This study suggests that WIN approaches—when supported by intentional design, adequate resources, and critical reflection—may foster a learning environment that is more inclusive, engaging, and meaningful for both teachers and students. While change can be difficult, the opportunity to align nutrition education with students' needs and lived experiences makes the effort both meaningful and significant.

### 4.1 Implications for School Health Policy, Practice, and Equity

Initial findings from teachers' experiences and perceptions suggest adopting a WIN curriculum may offer schools a valuable opportunity to address weight stigma, reduce weight‐based bullying, and potentially mitigate risk factors associated with disordered eating behaviors. Through the implementation testing process, teachers also helped to develop a curriculum that was responsive to students with limited food autonomy and sensitive to those who may be experiencing food insecurity. Teachers also felt that the WIN curriculum was exciting to teach and engaging for students because it was innovative and in‐line with how students wanted to learn about nutrition. To strengthen the impact that a WIN curriculum can have on student health outcomes, several priorities emerge. First, teachers identified their own limited knowledge about nutrition or weight inclusivity as well as access to professional development as challenges (Theme 1). These challenges could be addressed through support for WIN programming by school administrators in the form of professional development funds and assistance identifying professional development opportunities. Previous research demonstrates that for health education programming to be effective, it must be supported at all levels by school administrators, districts and states [23, 56, 57]. Additionally, curricular materials should include teacher notes, answers to frequently asked questions students may have, and options for adapting material to various classroom contexts. These types of built‐in curricular supports may help more health educators engage with new curricular approaches like WIN and feel confident doing so. Additional ways districts could support teachers include initiating district‐wide pilots of WIN curriculum where teachers in different schools could form mentoring structures and conduct peer observations to build community around a new WIN approach. Peer support can be extremely helpful to teachers with research showing that peer learning communities positively impact both teaching practice and student achievement through ongoing collaboration between teachers [58, 59].

### 4.2 Limitations

Several limitations for this study must be considered. Purposeful sampling of three teachers from one geographic area provided in‐depth insights but not generalizable findings or insights about patterns or trends. Multi‐state studies with diverse teacher samples collecting longitudinal data are needed to make broader claims about the ease of adoption and efficacy of WIN curricular materials. Furthermore, although the three teachers had varying familiarity with WIN concepts, they were all white females with substantial experience teaching health education. Findings may be different for teachers in different demographic groups or with fewer years of health education experience. Additionally, the focus on teacher experience does not capture student perspective. Further engagement is needed with additional schools and teachers across multiple grade levels and geographic areas.

## 5 Conclusions

This exploratory study suggests that nutrition education in high‐school health classes can be meaningfully re‐framed. Using teachers as partners, an eight‐lesson WIN curriculum was developed that emphasizes improved health and well‐being through sustainable behavior change and body acceptance. Teachers' experiences throughout the study revealed both challenges and opportunities inherent in implementing such an approach, requiring courage, adaptability, and resilience as they navigated a new paradigm. Furthermore, participant insights underscore the crucial need for on‐going WIN specific support, collaboration and shared learning among teachers and schools to facilitate the long‐term adoption and fidelity of the curriculum. Future research should build on these findings by evaluating both the intended and unintended outcomes on students' nutrition knowledge, attitudes, and health‐related outcomes.

## Funding

This work was supported by USDA Hatch funds.

## Ethics Statement

This study [CHRBSS (Behavioral): STUDY00002892] was reviewed and approved on January 16th, 2024 by the Chair of the IRB at the University of Vermont using the exempt procedures set forth under 45 CFR 46.104. While the project was exempt from IRB review, all researchers followed human subject protection regulations.

## Conflicts of Interest

The authors declare no conflicts of interest.

## Supporting information


**Data S1:** Supporting Information.

**Data S2:** Supporting Information.

**Data S3:** Supporting Information.

**Data S4:** Supporting Information.

**Data S5:** Supporting Information.

## Data Availability

The data that support the findings of this study are available from the corresponding author upon reasonable request.
