# Supplementary material for: Ditching Diet Talk: A Qualitative Study of Teachers Implementing Weight‐Inclusive Nutrition Curriculum in the High School Health Classroom
Source: J Sch Health. 2026 Apr 13;96:e70150. doi: 10.1111/josh.70150 (PMC13076096; doi:10.1111/josh.70150)
Supplement: Supplementary file 1 — Data S1: Supporting Information. [file JOSH-96-0-s005.docx]

WIN ~ Lesson Observation Tool

School Code: __________________________________

Date of Observation: ______________________________

Grade Level: _________ Number in Class: ___________ Duration of Class: ___________

Name of Lesson Observed: ______________________________

Observer Name:_________________________________

| **Question** | **Description** | **Yes** | **No** | **Comments** |
| --- | --- | --- | --- | --- |
| Overall, did the educator appear comfortable with implementing the material? | The educator appeared confident and did not appear nervous or uncertain about any of the material that they were implementing. |  |  |  |
| Overall, did the students appear comfortable with receiving the implemented material? | The students appeared interested and engaged throughout the lesson. |  |  |  |
| Specifically, were the students receptive to the nutrition messaging? | The students were actively engaged with the nutrition messaging and appeared interested. |  |  |  |
| Specifically, were the students receptive to the lesson activities? | The students appeared to enjoy the lesson activities. |  |  |  |
| Did the educator appear to be confused about implementing any of the material? | The educator may pause during the lesson or be unclear with the students about the content. |  |  |  |
| Did the students appear to be confused about any of the material implemented? | Students ask clarifying questions that would indicate that they are confused. Body language may also be used to determine confusion. |  |  |  |
| Were there any obvious points of contention in the classroom during the lesson? | Students and/or the educator displayed signs of disagreeing or being uncertain of the material being implemented. |  |  |  |
| Were there any “a-ha” moments with the students? | Students visibly become excited about the implemented material or acknowledge that “they get it”. |  |  |  |
| Overall, was the lesson clear? | Clarity could be in the delivery, the messaging, or the activities. |  |  |  |
| Did the lesson appear to impact the students? | Impact may be observed by student comment or body language. |  |  |  |
| Did the lesson run to the allotted time? | The lesson finished in the time alloted. |  |  |  |

What stands out the most to you as the observer of this lesson?

Areas done well:

Areas that could be improved:

Action Items Needed: (examples – further professional development, additional resources etc.)
