# Supplementary material for: Ditching Diet Talk: A Qualitative Study of Teachers Implementing Weight‐Inclusive Nutrition Curriculum in the High School Health Classroom
Source: J Sch Health. 2026 Apr 13;96:e70150. doi: 10.1111/josh.70150 (PMC13076096; doi:10.1111/josh.70150)
Supplement: Supplementary file 2 — Data S2: Supporting Information. [file JOSH-96-0-s003.docx]

Thank you for your hard work today! Please take a moment to answer the following questions about today’s lesson.

Name of Lesson:

1. How do you feel today’s lesson went? Explain why.
2. What components worked well?
3. What things were sticky for you?
4. What would you change to make the lesson better?
5. What kind of professional development do you think would help with this lesson?
6. What did/didn’t work well for your students?
