# Supplementary material for: Ditching Diet Talk: A Qualitative Study of Teachers Implementing Weight‐Inclusive Nutrition Curriculum in the High School Health Classroom
Source: J Sch Health. 2026 Apr 13;96:e70150. doi: 10.1111/josh.70150 (PMC13076096; doi:10.1111/josh.70150)
Supplement: Supplementary file 3 — Data S3: Supporting Information. [file JOSH-96-0-s004.docx]

The responses from the individual reflection questions completed at the end of each lesson will guide the post-pilot interview questions.

1. Has your thinking about nutrition, weight and health changed since you piloted the curriculum? If so, how?
2. Learning about weight inclusivity requires an un-learning of standard practices around nutrition, weight and health. Can you provide any examples from the lessons where you have had to un-learn/shift how you think?
3. Did you notice that you were thinking differently about how you use language around nutrition, weight and health? If so, can you provide some examples?
4. Overall, how do you think these lessons could be improved?
   1. For educators?
   2. For students?
5. If you were a teacher new to this concept, what kind of professional development or resources do you think would be most helpful?
6. What barriers or challenges so you see with this curriculum?
7. The Agency of Education has a list of transferable skills graduation proficiencies and performance indicators (i.e. communication, problem-solving, integrative thinking, citizenship and self-direction). How do you see these lessons providing students with these skills?
8. If our research team was to create an example performance assessment for this unit, what do you think that could look like?
