# Supplementary material for: Ditching Diet Talk: A Qualitative Study of Teachers Implementing Weight‐Inclusive Nutrition Curriculum in the High School Health Classroom
Source: J Sch Health. 2026 Apr 13;96:e70150. doi: 10.1111/josh.70150 (PMC13076096; doi:10.1111/josh.70150)
Supplement: Supplementary file 4 — Data S4: Supporting Information. [file JOSH-96-0-s002.docx]

1. What is your role as an educator? How long have you been teaching/How long have you been teaching health education?
2. Background/education/training:

- What is your educational background – undergrad/grad school?
- Do you have training specifically in health? Nutrition?

1. Professional development:

- How do you stay current in nutrition and weight-related topics that you teach?
- What are some nutrition and weight-related related resources that you regularly consult?
- Have you engaged in any nutrition or healthy body-focused professional development?

1. Curriculum:

- What informs your curricular decisions around nutrition and healthy bodies?
- How much nutrition/healthy bodies education do you usually do – weeks/class periods/hours?
- What is your approach to teaching about nutrition and healthy bodies?
  - Can you give me an example of an activity that you use to teach about nutrition or healthy bodies?
- Do you teach about BMI in your class?
  - If yes, could you share an activity that you do to engage students in the topic?
  - If no, why do you not teach about BMI?
- Do you teach about acceptance of body sizes differences? If so, how do you approach it?
- How do you define healthy eating?
- How do you teach about the benefits of healthy eating?
- How do you approach teaching about different foods and food choices?
- What is your approach to discussing unhealthy weight control practices and eating disorders?

1. How do you feel about piloting this curriculum? Are you excited, nervous, other?
2. Are there any nutrition topics that you are looking forward to teaching about? What about topics that you are hesitant or unsure to teach?
