# Supplementary material for: Ditching Diet Talk: A Qualitative Study of Teachers Implementing Weight‐Inclusive Nutrition Curriculum in the High School Health Classroom
Source: J Sch Health. 2026 Apr 13;96:e70150. doi: 10.1111/josh.70150 (PMC13076096; doi:10.1111/josh.70150)
Supplement: Supplementary file 5 — Data S5: Supporting Information. [file JOSH-96-0-s001.docx]

**WIN Education – Curriculum Development**

**Codebook**

| **Code** | **Definition** | **Application/Example** |
| --- | --- | --- |
| **Educational Approach** | | |
| Curriculum/resources used | - Specific materials consulted or used for class are mentioned | - National Health Education Standards - Michigan Model for Health - Internet resources - Continuing education |
| Weight normative/diet culture | - weight is an in important indicator of health - foods are referred to as ‘good’, ‘bad’, ‘healthy’, ‘unhealthy’ - BMI is used | - language is used or materials are mentioned that indicate a weight-normative approach - “100% orange juice is a better choice than sunny-d” |
| Weight inclusive/anti-diet culture | - we are all destined to be a different weight - focus on health behaviors - foods are not dichotomized - BMI is not used | - language is used or materials are mentioned that indicate a weight-inclusive approach - “remember that food is neither good, nor bad, it’s just food” |
| Basic nutrition concepts | - teacher discusses nutrition topics covered that are neither weight-normative nor weight-inclusive | - “we talk about food groups, we talk about nutrients” |
| **Curricular Features (Aspects)** | | |
| Constraints | - Lack of time, resources and/or professional development are identified as pedagogical challenges | - “I don’t have the time” - “There is no money for professional development” - “Health education is not a priority” |
| Uncertainty/Hesitation | - teacher expresses hesitation or lack of confidence when: - teaching new material for the first time - discussing implementation of WIN education - acknowledging student relationships with food | - educator acknowledges that students are triggered by certain activities and therefore they avoid talking about them - “I’m not sure how to answer those tough questions” - "I am terrified of giving you all a complex about food and making this a really negative experience." |
| Unlearning of standard practices | - teacher acknowledges a need for change - teacher identifies a shift in thinking - personal reflections on educational approach are expressed | - “students want this” - “I see how this requires a lot of unlearning and unpacking” - educator acknowledges that their current approach may be harmful to students |
| Personal influence | - teacher identifies the influence of their personal relationship to food | - “This is a journey. In my head I understand. But I’ve always struggled with eating” |
| Lesson Design | - lesson structure and pace are discussed - Teacher reflects on what worked well or needed improvement within lessons | - “you need to add connectors between lessons” - “timing of the activities was great” - “how would I do it different next time?” |
| **Curricular Opportunities** | | |
| Innovation | - teacher expresses excitement for the possibilities with this curriculum - skills-based curriculum is mentioned - teacher identifies areas within the curriculum to build out health equity - adaptation of teaching methods is discussed | - "You were able to take all of the theory of weight-inclusive nutrition and then actually make it a practical thing." - "My underlying thinking has honestly just been strengthened and reinforced." - SDOH and/or advocacy are named |
| Support and collaboration | - Professional development opportunities within the learning community are identified - Resources are identified that would be beneficial to educator learning around WIN principles - Teacher discusses current methods of support and collaboration or identifies a need for future support and collaboration | - professional learning communities - conversations with colleagues are mentioned or sharing of resources - “It would be great if we could collaborate with other teachers doing this kind of work” - “I would like more information on diet culture” |
| Cultivating relationships | - opportunities to build relationships with students and/or with others in the school community are discussed - teacher expresses investment in their role as an educator and to students | - “you have to know your students before you can talk to them about this” - student success is promoted - relationships with students are emphasized |
| **Effective Curricular Adoption** | | |
| Student autonomy & empowerment | - teacher discusses the ability of students to take control of their learning and make decisions to implement change in their lives/school | - “it's okay to eat something just because you want to eat something for pleasure or for fun" - "Many of them talked about weight inclusivity and how much they get out of it, which was awesome." |
| Lesson engagement | - teacher identifies student engagement in lessons | - “The students don’t want to talk about MyPlate. This is what they’ve been asking for” |
| Activity effectiveness | - teacher identifies lesson activities as being successful in student learning re WIN | - “students acknowledge that the negative effects of diet culture are real” - "the students seemed to get that nutrition is about more than just eating the most nutrient-dense foods." |
| Student questioning | - teacher acknowledges student questioning about WIN content | - areas of “stickiness” for students are identified i.e. diet culture - questions are asked about the dichotomization of food - “students were stuck on the calories in = calories out paradigm” |
| **Other** | | |
| Student driven feedback and requests regarding nutrition content | - students identify the need for alternative curriculum i.e. one with a weight-inclusive focus - students express feelings (positive or negative) about current curricular practices - students identify current curricular aspects are triggering for them | - “I have heard that students want more weight-inclusive material” - “Every semester there’s one or two who come to me and say they can’t do the assignment – it's triggering” |
